# The songs of male pied flycatchers: exploring the legacy of the fathers

**DOI:** 10.7717/peerj.5397

**Published:** 2018-08-01

**Authors:** Antonieta Labra, Helene M. Lampe

**Affiliations:** Centre for Ecological and Evolutionary Synthesis (CEES), Department of Biosciences, University of Oslo, Oslo, Norway

**Keywords:** Song duration, Repertoire size, *Ficedula hypoleuca*, Song tutor, Open-ended learner, Heritability, Additive genetic variance

## Abstract

Singing is a key element of songbirds’ behavioral repertoire, particularly for males, which sing during the breeding season to defend resources against other males and to attract females. Different song traits may convey honest information about males’ qualities or conditions, which may be used by females to select their mates. Traits under strong sexual selection have an important component of additive genetic variation (i.e., the main genetic inheritance from parents), and so relatively high heritability; therefore, it can be expected that song traits also do. Although the act of singing is an innate behavior, and thus, genetically determined, songbirds need to learn their songs and therefore the genetic contribution to song traits may be reduced by the effect of environmental factors. We tested this hypothesis in seven song traits recorded in the long-distance migratory bird, the pied flycatcher (*Ficedula hypoleuca*). From a 23-year database (1992–2015), we obtained songs for 28 father–son pairs, and for each song trait we applied parent–offspring regressions to estimate heritability. The type of syllables sung are learned from tutors, and here we also determined the cultural contribution of fathers to the song repertoires of their sons, by quantifying the percentage of syllables that sons shared with their fathers, and compared this with what sons shared with other males in the population (e.g., neighbors). The heritabilities of song traits were highly variable (ranging from −0.22 to 0.56), but most of these were around zero and none of them were significant. These results indicate that the seven song traits are most likely determined by environmental factors. Sons shared more syllables with their fathers than with neighbors (21% vs. 3%), suggesting that fathers are important song tutors during the nestling period. We conclude that there is a cultural inheritance from fathers to their sons’ syllable repertoires, but there is no strong evidence for a genetic contribution of fathers to the seven song traits studied.

## Introduction

Singing is a fundamental component of the songbirds’ behavioral repertoire that evolves under sexual selection (Catchpole & Slater, 2008). In species from temperate areas, mainly males sing, especially during the breeding season (Slater & Mann, 2004), to defend resources against other males and/or to attract females (Catchpole, 1987; Searcy et al., 2014). Females may select a male based on the information encoded in his songs, as different song traits may convey honest information about male characteristics or conditions (Nowicki, Peters & Podos, 1998; Gil & Gahr, 2002), such as their phenotypic (Richner, 2016) or genetic quality (Ferrer et al., 2015), and age (Espmark & Lampe, 1993; Kipper & Kiefer, 2010; but see Motes-Rodrigo, Labra & Lampe, 2017). In addition, song traits may reveal males’ physiological states (e.g., developmental stress (Nowicki et al., 2000; Spencer et al., 2005b), immune condition (York et al., 2016), body condition (Lampe & Espmark, 1994; Mountjoy & Lemon, 1996), or parasite levels (Spencer et al., 2005a)), as well as some of their behavioral characteristics (e.g., quality of the territories (Lampe & Espmark, 2003; Ritschard & Brumm, 2012), ability to provide paternal care (Buchanan & Catchpole, 2000; but see Rinden et al., 2000), or breeding experience (Lampe & Espmark, 1994; Motes-Rodrigo, Labra & Lampe, 2017)). Thus, it is not surprising that the fitness of males may be associated with song traits, such as song duration (Searcy et al., 2010), versatility (Järvi, 1983; Lampe & Espmark, 2003), or complexity (Soma & Garamszegi, 2011, but see Byers & Kroodsma, 2009; Potvin et al., 2015).

Sexual traits seem to be more determined by the additive genetic variance (i.e., the main genetic inheritance from parents) than non-sexual traits (Pomiankowski & Møller, 1995; Bakker, 1999). This conclusion is based on metrics that compute the genetic vs. the environmental determination of a trait, such as the coefficient of additive genetic variance (i.e., mean-standardized index of additive genetic variance of a trait; Houle, 1992), and heritability (i.e., proportion of phenotypic variance attributable to genetic variance; Falconer & Mackay, 1996); higher values indicate higher genetic determination on the studied traits. It is therefore expected that song traits, as important sexual traits (Catchpole & Slater, 2008), have relatively high heritability and coefficient of additive genetic variance. This is, however, not very likely as song development is a complex process in songbirds. Singing is an innate behavior, although the acquisition of the correct song elements requires learning (Hultsch & Todt, 2004; Beecher, 2017), and the lack of a song tutor can produce aberrant songs, without the proper element characteristics of the species’ song (Slater, 1989). In addition, when tutors are present, even if song learning has a genetic predisposition for learning conspecific songs (Mundinger & Lahti, 2014), the development of other song characteristics (e.g., duration, repertoire size) can be modulated by different factors (e.g., early nutritional condition, Nowicki, Peters & Podos, 1998), which may reduce the genetic contribution of parents to these traits.

Few studies have quantified the genetic component of song traits. The existing data, mainly obtained under controlled laboratory conditions, indicate that heritability is highly variable across song traits and species. In the zebra finch, *Taeniopygia guttata,* a significantly high heritability was found in the proportion of unique syllables in the song (Woodgate et al., 2014), and in the song rate (Houtman, 1992), while traits such as the repertoire size or song duration had low, but significant heritabilities (Forstmeier et al., 2009). In contrast, in the canary, *Serinus canaria*, song duration and repertoire size showed a high heritability, while the heritabilities of the song activity and consistency were not significant (Trösch et al., 2017). Non-significant heritabilities were also found in song amplitude (Ritschard & Brumm, 2011) and phrase length (Woodgate et al., 2014) in the zebra finch, and in song duration in the splendid fairy-wren, *Malurus splendens* (Greig, Taft & Pruett-Jones, 2012).

Here we aimed to explore, under natural conditions, the heritability and coefficient of additive genetic variance of seven song traits in the pied flycatcher (*Ficedula hypoleuca*), some of them known to be selected by females (see below). This species is a long-distance migrant that breeds in temperate forests across large parts of Europe and Western Asia and winters in sub-Saharan, tropical West Africa (Lundberg & Alatalo, 1992; Ouwehand et al., 2016). Pied flycatchers, therefore, can experience important variation in environmental conditions that may modulate significantly their song development, and thus reduce the genetic contribution to song traits.

Upon arrival at the breeding site, male pied flycatchers sing to attract females, and females prefer males with more complex songs (e.g., more versatile and with larger repertoire size; Lampe & Sætre, 1995; Sirkiä & Laaksonen, 2009). After mating, males sing significatively less (Lundberg & Alatalo, 1992; Espmark & Lampe, 1993), except the polyterritorial males that start to sing at a second nest box to attract another female (Slagsvold et al., 1992). Therefore, nestlings may have few opportunities to learn song elements (i.e., syllables) from their fathers during this sensitive period for vocal learning (Eriksen, Lampe & Slagsvold, 2009). To clarify if there is a cultural inheritance from fathers to their sons’ song development, we determined the syllables shared by sons and their fathers.

In summary, we studied inheritance in pied flycatcher songs, determining the genetic contribution to seven song traits. We predicted that heritabilities and coefficients of additive genetic variance of pied flycatcher song traits would be low, because environmental factors may play a stronger role on song development. Additionally, we assessed the fathers’ cultural contribution by quantifying the shared syllables between fathers and sons. Because fathers reduce singing time dramatically after mating, we hypothesize that it is unlikely that fathers tutor their sons, and thus, the father’s cultural contribution to a son’s song repertoire would be small, or inexistent. Most likely, sons will share a similar amount of syllables with their fathers as with other adult males.

## Materials and Methods

### Song collection and analyses

The study was conducted at Sinober (59°59′N, 10°38′E; 150–206 m asl), NW Oslo, Norway. The ca. 72 ha area is a mixed coniferous forest where from 1985 around 225–275 wooden nest boxes were erected on trees, 1.5 m above the ground, which are renewed when worn-out. These nest boxes are 15 × 17 × 27 cm and have an entrance hole of 3.5 cm in diameter.

During the breeding seasons of 1992–2015, songs were recorded just after the arrival of the males and before mating. After the recordings, the new males were captured, marked with numbered and color bands, and then released. Nestlings were banded when they were 13 days old to identify them in the next breeding season, usually the following year, when their singing would be recorded.

Songs were recorded between 07:00 and 12:00 am using Telinga parabolic microphones (Telinga, Tobo, Sweden). Until 2006, recordings were made with a TC-D5 Pro II Sony cassette recorder (Sony Corporation, Tokyo, Japan), which were later digitalized and stored as .WAV files using Raven Pro 1.4 (Cornell Laboratory of Ornithology, Ithaca, NY, USA). From 2007, songs were directly obtained using digital recorders, Sound Devices 702 (Sound Devices, LLC, Reedsburg, WI, USA) or Marantz PMD661 MK11 (D&M Holdings Inc., Tokyo, Japan), at a sample rate of 44.1 kHz.

We obtained song recordings for 28 father–son pairs, for which we did not obtain genetic samples, due to logistic reasons. For this population however, data indicate that only 4–7% of nestlings are from extra-pair copulations (Lifjeld, Slagsvold & Lampe, 1991; Ellegren et al., 1995), and therefore, no more than two sons in our sample might be paired with a social, rather than with a genetic, father. We also obtained song recordings for the neighbors of these father–son pairs. Because we were testing for cultural transmission, we included males that defended a nest box within 100 m of the focal nest. This distance ensured that songs would not suffer a significant sound degradation within the nest box for nestlings to be able to hear the neighbor songs, if he sang (see Lampe et al., 2004, 2007), and eventually, to learn some syllables. Because of this distance restriction, we only included 15 male neighbors.

**Figure 1:**
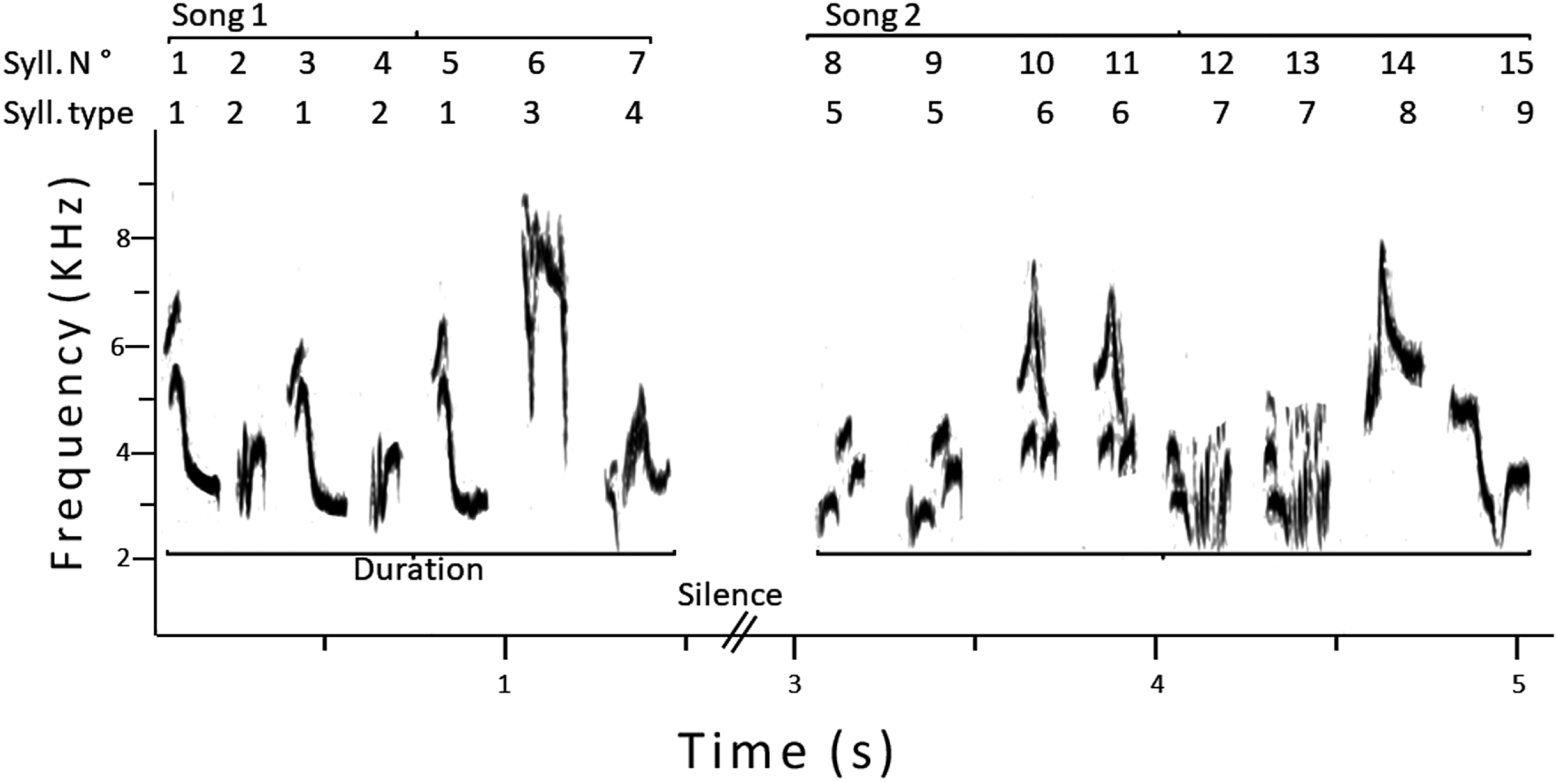
Sonogram of two consecutive songs of a male pied flycatcher. The numbering of syllables is shown, and how the song duration was measured. In this example, the total number of syllables was 15, the song repertoire size was nine, the sample versatility was 0.6 and the average song versatility was 0.59.

For each male, we analyzed a sample of 25 consecutive songs, using Raven. Considering previous studies on song analyses in this species (Espmark & Lampe, 1993; Motes-Rodrigo, Labra & Lampe, 2017), we measured the following variables (see Fig. 1 for an example): (1) song duration, to the nearest 0.01 s; (2) total number of syllables per song; (3) total number of different syllable types per song; (4) sample repertoire size, that is, the total number of different syllables in the whole sample (25 songs); (5) total number of syllables in the whole sample; (6) song versatility: the ratio between three and two, that is, total number of different syllables per song/total number of syllables per song; (7) sample versatility: the ratio between five and four, that is, sample repertoire size/total number of syllables in the whole sample. To reduce variation in syllable differentiation, only one person (Antonieta Labra) performed the song analyses using the same criteria for all males.

By visual inspection of sonograms (Mason, Shultz & Burns, 2014), we determined the shared syllables of each pair of males (e.g., father–son, son–neighbor, father–neighbor); this was a consensus between authors. Then, we calculated the percentage of syllables shared by males, and the DICE sharing coefficient: *C_D_* = 2 * N° of shared syllables/Male 1 (e.g., father) repertoire size + Male 2 (e.g., son) repertoire size (Eriksen, Slagsvold & Lampe, 2011). *C_D_* can reach values between 0 (no sharing) and 1 (identical repertories). We checked if any of the syllables shared by the father–neighbor pairs were also shared by sons. If so, we excluded those syllables in common to all these males, because most likely nestlings learned these syllables from fathers rather than from neighbors. Finally, to determine if the proximity between males modulates the song repertoires, we compared the syllables shared by six fathers with their neighbors and non-neighbors, including only male pairs recorded in the same year to minimize differences between years.

We obtained authorization to perform field work from Miljø Direktoratet (Norway) for each year.

### Statistical analysis

The heritability of each song trait was obtained using parent–offspring regressions, that is, the linear regression of son on father trait. We used twice the slopes of the regression estimates to get the heritability, *h*^2^ (Falconer & Mackay, 1996); the standard error of the regression slope was also doubled. We also measured the coefficient of additive genetic variance, √*V_A_*/mean (Houle, 1992), where *V_A_* (additive genetic variance) is equal to *h*^2^ * *V_P_*, and *V_P_*, is the phenotypic variance of the trait, such as the repertoire size. For each song trait studied, the calculations of the coefficients of additive genetic variance were made using the mean and variance from fathers.

To determine if son’s song traits were affected by neighbors, we performed simple regressions between neighbors and sons, on the seven song traits.

In this population the breeding experience, more than age, modulates the song traits (Motes-Rodrigo, Labra & Lampe, 2017), and in our sample, seven fathers had breeding experience when they became fathers of the sons incorporated in the study. Therefore, we also performed an Analysis of Covariance (ANCOVA) to recalculate *h*^2^ incorporating the father breeding experience. The ANCOVA results showed the same trends as those obtained with the simple regressions, and therefore, we only present the latter.

Our sample was not free of pseudoreplication, as three sons became fathers (one of these became a father of two sons), and in four cases two males shared a father (i.e., they were brothers). Due to the overlap between these two situations, five pairs caused this pseudoreplication, and new analyses were done excluding these pairs. We did not perform separate regression analyses of siblings sharing a father, due to the low sample size (*n* = 5).

To determine if the percentage of shared syllables and the DICE coefficient could be associated with father’s or son’s song traits, we applied Simple Pearson Correlations, using the 28 father–son pairs. Finally, we compared the proportion of syllables shared and the DICE coefficients between the different pairs of males (e.g., father–son and son–neighbor) using *t*-tests for repeated measurements.

Unless otherwise indicated, in general we discuss data referring to the 28 father–son pairs, as trends were similar before and after removing the causes of pseudoreplication. Data are presented as mean ± SE.

## Results

**Figure 2:**
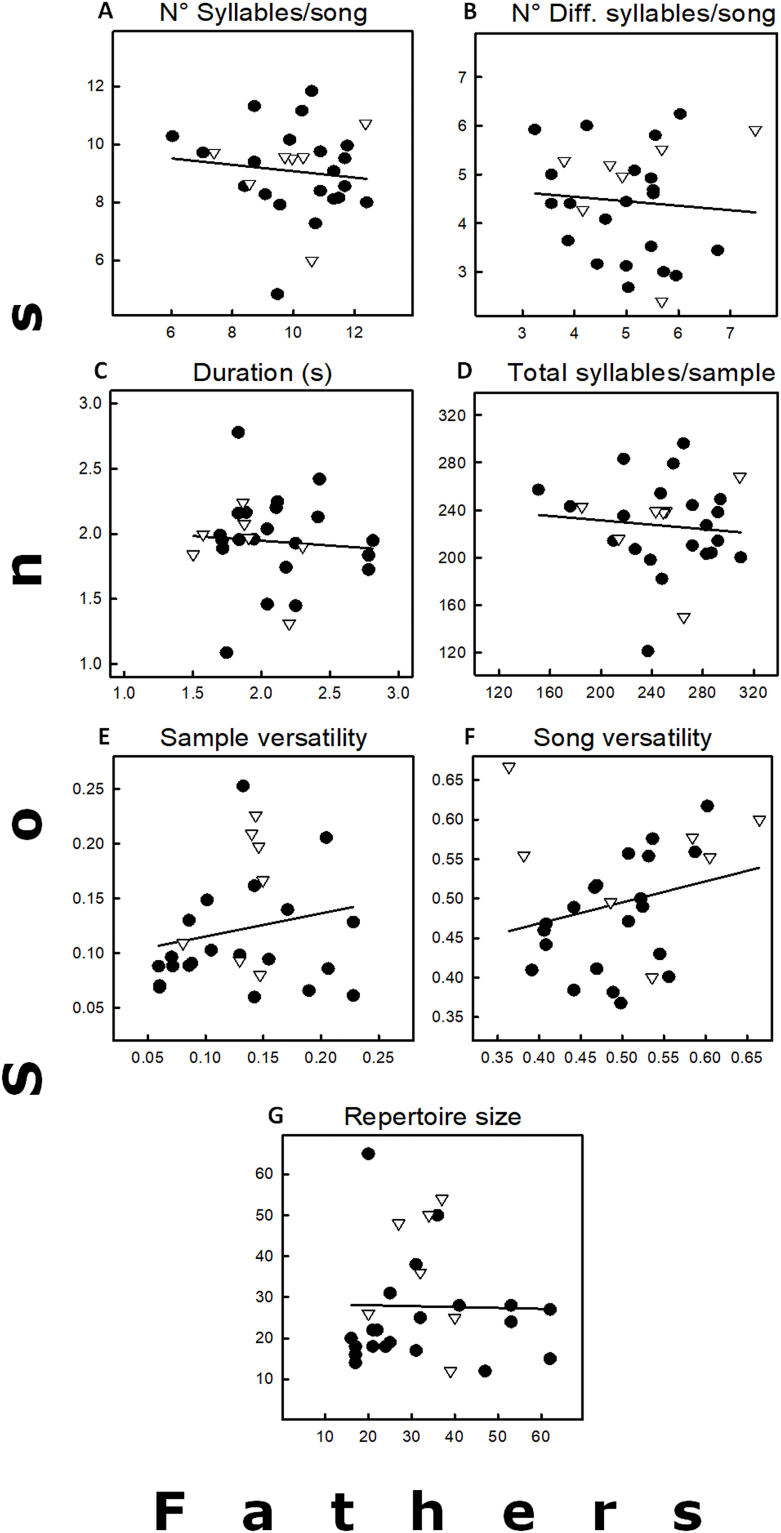
Relationships between fathers and sons for seven song traits, including the regression lines. (A) Total number of syllables by song. (B) Total number of different syllables by song. (C) Song duration (s). (D) Total number of syllables by sample. (E) Sample versatility. (F) Song versatility. (G) Repertoire size. For statistics see Table 1. Symbols: Δ = pairs in which the father had previous breeding experience. • = Fathers without breeding experience.

The average values of the seven variables recorded for the 28 father–son pairs are in Fig. 2, and the corresponding heritability values in Table 1. These heritability estimates were highly variable (−0.45 to 0.53), and none of them significant (Table 1). Only two variables (song and sample versatility) showed positive heritability values (*h*^2^ = 0.53 and 0.42, respectively), but they had very low coefficients of additive genetic variance (Table 1). The other five variables with negative heritability estimates were considered equal to 0 (see Réale & Festa-Bianchet, 2000), as well as their coefficients of additive genetic variance. The high variance of all the heritability estimates, however, does not allow the exclusion of a small heritability. Similar trends were obtained when analyses were done excluding the five father–son pairs that caused pseudoreplication. The main difference was that duration had a positive *h*^2^, but it was close to zero (Table 1). Finally, none of the simple regressions between neighbors and sons for the five song traits were significant (*p* > 0.05; sample size = 15; data not shown as regressions were similar as those found in Fig. 2).

**Table 1:** Descriptive statistics, mean ± SE and variance (*V_P_* = phenotypic variance)^1^, of seven song traits of pied flycatchers.

	Fathers		Sons				
	Mean ± SE	*V_P_*^1^	Mean ± SE	*V_P_*^1^	*h*^2^ ± SE	CV_*A*_	*R*^2^ (*p*)
Total N° syllables per song	10.03 ± 0.30	2.563	9.07 ± 0.29	2.299	−0.22± 0.37	0	0.0136 (0.56)
	**9.90 ± 0.35**	**2.802**	**9.12 ± 0.30**	**2.131**	−**0.10 ± 0.38**	**0**	**0.0031 (0.80)**
Total N° diff. syllables per song	5.00 ± 0.19	1.000	4.45 ± 0.21	1.234	−0.18 ± 0.43	0	0.0068 (0.68)
	**4.99 ± 0.22**	**1.090**	**4.49 ± 0.24**	**1.296**	−**0.05 ± 0.48**	**0**	**0.0006 (0.91)**
Repertoire size	32.21 ± 2.54	181.286	27.79 ± 2.64	195.656	−0.05± 0.41	0	0.0005 (0.91)
	**31.65 ± 2.69**	**166.783**	**29.35 ± 3.05**	**214.146**	−**0.07 ± 0.49**	**0**	**0.0010 (0.89)**
Total N° syllables in sample	249.79 ± 7.53	1588.397	226.82 ± 7.16	1437.115	−0.19 ± 0.37	0	0.0094 (0.62)
	**246.26 ± 8.66**	**1726.383**	**227.96 ± 7.61**	**1332.134**	−**0.05 ± 0.38**	**0**	**0.0009 (0.89)**
Song versatility	0.50 ± 0.01	0.006	0.49 ± 0.29	0.006	0.53 ± 0.40	0.11	0.0637 (0.20)
	**0.50 ± 0.02**	**0.006**	**0.50 ± 0.02**	**0.007**	**0.56 ± 0.44**	**0.12**	**0.0711 (0.21)**
Sample versatility	0.13 ± 0.01	0.003	0.12 ± 0.01	0.003	0.42 ± 0.41	0.27	0.0384 (0.32)
	**0.13 ± 0.01**	**0.002**	**0.13 ± 0.01**	**0.003**	**0.47 ± 0.50**	**0.26**	**0.0402 (0.36)**
Duration (s)	2.06 ± 0.07	0.124	1.94 ± 0.06	0.113	−0.15 ± 0.37	0	0.0059 (0.70)
	**2.02 ± 0.07**	**0.122**	**1.94 ± 0.06**	**0.088**	**0.08 ± 0.37**	**0.05**	**0.0024 (0.82)**

**Notes:**

Data for 28 father–son pairs. The second line (in bold) of each variable has the results of the analyses removing five pairs with pseudoreplication (*n* = 23). *h*^2^ = estimations of the heritability, obtained from father–son regressions, and the coefficient of determination (*R*^2^) of regression and the corresponding probability (*p*). CV_*A*_, coefficient of additive genetic variance. Negative heritability estimates were considered equal to 0, as well as the coefficients of additive genetic variance. Calculations were made considering the mean and the phenotypic variance of the fathers.

1 *V_P_* = N * (SE)^2^, where N = sample size and SE = standard error, the standard deviation of the sampling distribution of the trait.

**Figure 3:**
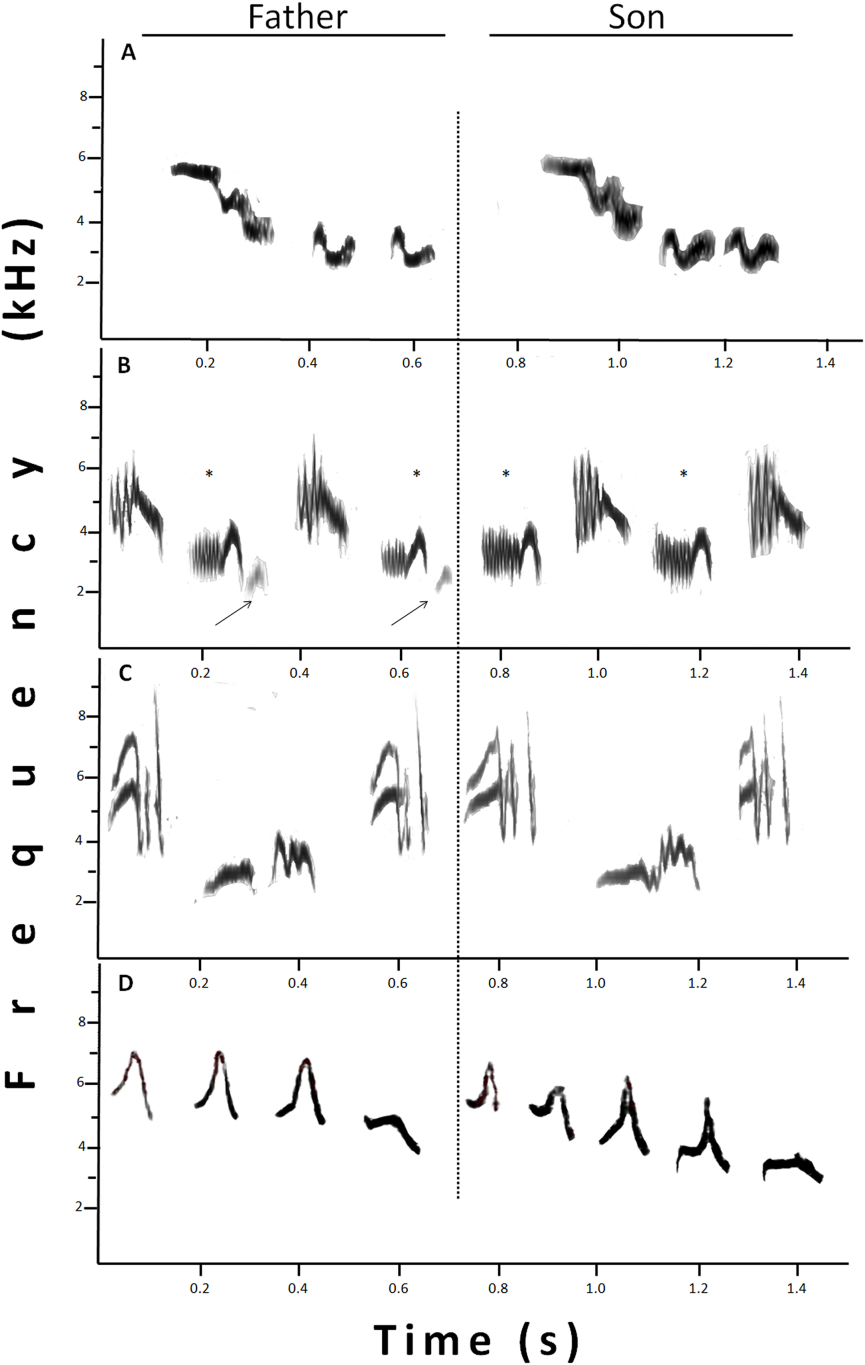
Sonograms that show different examples of shared syllables between fathers (at the left) and sons (at the right). (A) Identical syllables shared. (B) Example of “missing part.” One syllable, marked with *, was similar between father and son, since the son syllable lacks the terminal part present in the father’s syllable (indicated by the arrow). (C) The two syllables sung by the son were only similar, and not identical, to those sung by the father, however, the high resemblance in a motif, made them considered as shared. (D) Father and son showing an imitation of a willow warbler. In this case, the whole set was considered one shared syllable.

Considering the 28 father–son pairs, we found that sons shared on average 20.70 ± 3.20% (min–max: 0–64%) of their song repertoire with their fathers (for an example see Fig. 3A), but had a low sharing coefficient, *C_D_* = 0.17 ± 0.02 (min–max: 0–0.49). In a few cases (<1%), the assigned shared syllables were not identical, but they showed a high resemblance. One of those cases was when part of a syllable was “missing” (Fig. 3B) and the other case was when the syllables were not completely similar (Fig. 3C), but in both cases, syllables showed high similarities by conforming to a motif, that is, syllables linked into structural units (Lampe & Espmark, 1994). Finally, there was one unique case: a father–son pair shared part of the aggressive type A song of willow warblers (Fig. 3D; for examples of willow warbler songs see Järvi, Radesäter & Jakobsson, 1980). The percentage of syllables shared by the father–son pairs correlated significantly with some of the father’s song traits: total number of syllables per song (*r* = 0.43; *p* = 0.021), total number of different syllables per song (*r* = 0.55; *p* = 0.002), repertoire size (*r* = 0.40; *p* = 0.037) and total number of syllables per sample (*r* = 0.46; *p* = 0.014). In contrast, the DICE sharing coefficient only showed a significant correlation with a single father’s song trait: total number of different syllables per song (*r* = 0.43; *p* = 0.024).

**Figure 4:**
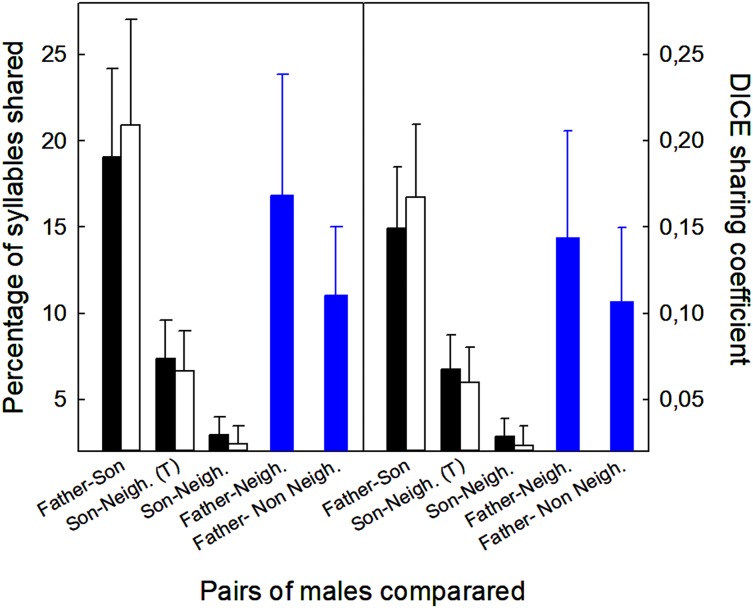
Values of the proportion of syllables shared between different males (fathers, sons, neighbors, non-neighbors), and the DICE coefficients. Mean + standard error of the proportion of syllables shared and DICE sharing coefficients of five types of male pairs compared. Comparisons include all 15 sons with a neighbor recorded within 100 m of the nest box (▪) and 12 sons after removing three pairs that shared the same neighbors (□). Sons were compared with fathers and neighbors. The figure shows the values including all the syllables shared with the neighbors (T = total), and excluding those that were shared by sons, fathers and neighbors (see Material and Methods for more explanations). Comparisons for fathers (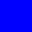) were obtained from six fathers that were compared with neighbors and non-neighbors.

Sons shared more syllables with their fathers than with their neighbors, regardless of whether we included or excluded those syllables in common among sons, neighbors, and fathers (Fig. 4). Analyses excluding three pairs that shared neighbors revealed the same trends (*t*-tests, *p* < 0.05; Fig. 4). Neighbors showed more similarities with fathers than with sons (12 vs. 3%, % syllables shared: *t*_12_ = 2.61, *p* < 0.02; 0.11 vs. 0.02, DICE coefficient: *t*_12_ = 2.69, *p* < 0.02). Finally, fathers shared similar number of syllables with neighbors and non-neighbors (Fig. 4; % syllables shared: *t*_6_ = 0.98, *p* > 0.05; DICE coefficient: *t*_6_ = 0.61, *p* > 0.05).

## Discussion

The seven song traits studied in pied flycatchers showed high variations in their heritability estimates and coefficients of additive genetic variance. The traits known to be attractive for females, song and sample versatility (Lampe & Sætre, 1995; Sirkiä & Laaksonen, 2009), showed a relatively high heritability (*h*^2^ = 0.53 and 0.42), but had very low coefficients of additive genetic variance, and were not significant. The other variables had negative estimates, and thus the best estimate for their heritabilities and coefficients of additive genetic variance was 0 (Réale & Festa-Bianchet, 2000). The high variances of the heritability estimates, however, do not allow us to completely rule out, some small heritabilities. Our data suggest, however, that the genetic additive variance of these song traits is small, and that most likely, environmental factors are playing a more relevant role in modulating these traits. In addition, the negative heritability estimates also suggest that these song traits were not culturally influenced by the genetic or social father (in case of extra-pair paternity).

Vocal learning is usually restricted to a sensitive period early in life, except in the open-ended learners, such as the pied flycatcher (Eriksen, Slagsvold & Lampe, 2011, and references therein), which can modify their songs throughout their life (Catchpole & Slater, 2008). Such species may show high variation in song traits because individuals can be exposed to different and more variable environmental conditions which can strongly modulate their song development. Thus, it is reasonable to expect less genetic influence in their songs. Our study included individuals recorded over 23-year period, during which the environmental conditions varied in the breeding and wintering areas, as well as along the migratory route (e.g., food availability; Cadahía et al., 2017). Therefore, fathers and sons may have experienced different environmental constraints that could affect their conditions (Samplonius et al., 2016), and therefore, song traits (Alatalo, Glynn & Lundberg, 1990; Ritschard & Brumm, 2012). In fact, the different song traits had very high variances, reflecting a high variability across individuals.

Variation in environmental conditions may not be the only explanation for our heritability results; at least four other factors may be involved. (1) Extra-pair copulations. Our assumption was that father–son pairs were genetically related, but extra-pair copulation is frequent in some populations of pied flycatchers (Gelter & Tegelström, 1992; Brün et al., 1996; Moreno et al., 2013, 2015), which may cause an underestimation of the heritabilities (Keller et al., 2001). In the studied population, however, extra-pair copulation is relatively low for nestlings, 4–7% (Lifjeld, Slagsvold & Lampe, 1991; Ellegren et al., 1995). Therefore, even if two individuals (7% of the sample) were compared with a social and not the genetic father, this would explain very little of the obtained heritability values. (2) Other associated variables. Song traits may correlate with other characters selected by females (e.g., body condition, morphology; Sirkiä & Laaksonen, 2009; Darolová et al., 2012), and that are inherited and less affected by the environmental conditions. For example, in pied flycatchers the sample versatility and repertoire size correlate with plumage color (Lampe & Espmark, 1994), a characteristic that is partly heritable (Slagsvold & Lifjeld, 1992). Females prefer brightly colored males (Sætre, Dale & Slagsvold, 1994), and these males seem to invest more in reproduction (Järvi et al., 1987), that is, they are better at feeding the young (Sætre, Fossnes & Slagsvold, 1995; but see Rinden et al., 2000). Therefore, under natural conditions, females may prioritize the information assessed from sources other than song traits, which could be more reliable indicators of a male with good genetic quality (Sirkiä & Laaksonen, 2009). (3) Song sample size: 25 songs for each individual may not be enough to characterize certain aspects of male songs (e.g., repertoire size; Espmark & Lampe, 1993; Eriksen, Slagsvold & Lampe, 2011), and therefore, to estimate heritability. Eriksen, Slagsvold & Lampe (2011) indicated, however, that a sample of 25 songs would be large enough to cover a sufficient proportion of the males’ repertoires to reveal variation and temporal changes in song traits, probably comparable to what prospecting females get to hear during visits to different males to select a mate, the main function of pied flycatchers’ songs. Thus, 25 songs may be an adequate sample to estimate heritability, as perceived by females, but this should be tested. (4) Small sample size. Depending on the number of father–son pairs considered, four or five out of seven of the heritability estimates were negatives, and considering that *h*^2^ range from 0 to 1, negative values indicate, most likely, estimation errors associated with a small sample size. Our data showed large variances, determining that *h*^2^ estimates were out of the expected range (0–1). Considering that small sample sizes can determine inaccurate heritability estimates (Falconer & Mackay, 1996), our heritability results should be taken cautiously.

The song repertoire of sons seems to be modulated by their fathers regardless of whether they are genetic or social fathers (Eriksen, Lampe & Slagsvold, 2009). In fact, sons shared 21% of the syllables with their fathers, although the DICE sharing coefficient showed a relatively low sharing (0.17%). Sons also shared some few syllables with neighbors (3%), although this was significantly less than what they shared with their fathers. Therefore, what is sung (i.e., syllables types), depends to a large extent on cultural transmission from fathers. This does not preclude that individuals learn syllables from other males later in life (Eriksen, Slagsvold & Lampe, 2011). This may explain why fathers shared in average 14% of their repertoires with other males, independently of the proximity of their territories. It is unclear, however, how fathers (or neighbors) contribute to a nestlings song development, considering that males reduce their general singing activity after mating (Espmark & Lampe, 1993). Fathers are occasionally observed to sing a song before entering the nest box to feed their nestlings (H. M. Lampe, 2004, personal observations), which would provide an opportunity for nestlings to develop their songs. Nestlings may also learn songs while males sing at dawn (not recorded in this study). Dawn singing seems to be more versatile (Vabishchevich, 2011), and in the closely related species, *F. albicollis*, males still exhibit some low-level singing at dawn while they have nestlings (Pärt, 1991). Considering the few opportunities that nestlings of pied flycatchers seem to have to learn songs, it may be possible that they require a short exposure to songs to learn, as has been documented for the nightingale, *Luscinia megarhynchos* (Todt & Geberzahn, 2003; Hultsch & Todt, 2004). In fact, 10- to 12-day-old pied flycatchers discriminate between songs of conspecifics and heterospecifics (Wheatcroft & Qvarnström, 2017), supporting the idea that males start their song development while they are still nestlings. Remarkably, both the percentage of shared syllables and the DICE coefficient correlated positively with the total number of different syllables that fathers sang per song, suggesting that nestlings learn more syllables when their fathers have more versatile songs.

The singing activity of pied flycatchers in areas other than the breeding grounds, such as the stopover areas and the wintering grounds, seems to be rare (Sorensen, Jenni-Eiermann & Spottiswoode, 2016; J. Ouwehand, 2006, personal communication). Even if pied flycatchers do not sing at the wintering grounds, they may learn new song elements from species that are singing, such as the willow warbler (*Phylloscopus trochilus*; Sorensen, 2014), which might explain the presence of willow warbler song elements in the pied flycatcher songs (Vabishchevich, 2012; present study). In our study, however, it is unlikely that both males of the father–son pair that shared willow warbler song elements have learned those syllables independently, either in the wintering ground, on migration, or at the breeding grounds. It is more parsimonious that the son had learned those song elements from its father when he was a nestling, indicating a cultural inheritance from the father. If both males had learned this in the breeding area, it would be expected that other individuals in our study would sing willow warbler syllables, particularly the neighbor of this father–son pair, which was not the case.

## Conclusions

Attempt to unravel the father’s contribution to the son’s song development without experimental manipulation is a challenging task because there are many uncontrolled factors that can affect the process. The results, however, can provide a more realistic picture of the phenomena under analysis. In our study, data from individuals recorded across 23 years indicate that the paternal inheritance of song development in their sons is mainly cultural; sons learn syllables from their fathers. The other song traits studied are determined more by environmental factors than genetic inheritance.

## Supplemental Information

10.7717/peerj.5397/supp-1 Supplemental Information 1 Raw data of song variables.

## Additional Information and Declarations

### Data Availability

The following information was supplied regarding data availability: The raw data are provided in a Supplemental File.
